# Exogenous glycosaminoglycans coat damaged bladder surfaces in experimentally damaged mouse bladder

**DOI:** 10.1186/1471-2490-5-4

**Published:** 2005-03-23

**Authors:** Kimberly D Kyker, Jean Coffman, Robert E Hurst

**Affiliations:** 1 Department of Urology, Oklahoma University Health Sciences Center, 940 S.L. Young Blvd, Oklahoma City, OK, 73104, USA

## Abstract

**Background:**

Interstital cystitis is often treated with exogenous glycosaminoglycans such as heparin, chondroitin sulphate (Uracyst), hyaluronate (Cystistat) or the semi-synthetic pentosan polysulphate (Elmiron). The mechanism of action is presumed to be due to a coating of the bladder surface to replace the normally present chondroitin sulphate and heparan sulphate lost as a result of the disease. This study used fluorescent labelled chondroitin sulphate to track the distribution of glycosaminoglycans administered intravesically to mouse bladder that had been damaged on the surface.

**Methods:**

The surfaces of mouse bladders were damaged by 3 mechanisms – trypsin, 10 mM HCl, and protamine sulphate. Texas Red-labeled chondroitin sulphate was instilled into the bladders of animals with damaged bladders and controls instilled only with saline. Bladders were harvested, frozen, and sectioned for examination by fluorescence.

**Results:**

The normal mouse bladder bound a very thin layer of the labelled chondroitin sulphate on the luminal surface. Trypsin- and HCl-damaged bladders bound the labelled chondroitin sulphate extensively on the surface with little penetration into the bladder muscle. Protamine produced less overt damage, and much less labelling was seen, presumably due to loss of the label as it complexed with the protamine intercalated into the bladder surface.

**Conclusion:**

Glycosaminoglycan administered intravesically does bind to damaged bladder. Given that the changes seen following bladder damage resemble those seen naturally in interstitial cystitis, the mechanisms proposed for the action of these agents is consistent with a coating of damaged bladder.

## Background

Interstitial cystitis (IC) is being re-examined within a broader context of chronic pelvic pain and pelvic floor dysfunction with the result that it is being recognized that the prevalence of IC is much higher than previously estimated [1,2]. It has long been evident that, whatever the cause, IC is a disease of the urothelium and the symptoms are triggered by urine [3]. In general, the bladder exhibits thinning and denudation of urothelium and distinctive changes in the muscle [4], and there is very strong evidence that the urothelium fails to differentiate properly in IC [5,6]

One of the main therapeutic approaches is so-called "glycosaminoglycan (GAG) replacement therapy." This is partly based upon earlier observation that the so-called "GAG Layer" is lost or damaged in the interstitial cystitis bladder [6,7]. The "GAG Layer" is a complex set of proteoglycans and glycoproteins on the bladder surface has been isolated and characterized from cow and human bladder [8-11]. Whether or not glycosaminoglycans actually restore impermeability of the bladder in interstitial cystitis is controversial [12-15]. Nonetheless, many patients respond to intravesical instillations of pentosan polysulphate (Elmiron) [16], hyaluronate (Cystistat) [17], chondroitin sulphate (Uracyst) [18]or heparin [19].

In this paper, we use three different models of bladder damage to determine whether GAG instilled intravesically actually binds preferentially to damaged bladder. We prepared a fluorescent-labeled chondroitin sulphate molecule by covalently attaching Texas Red to the reducing terminus of the GAG chain and then observing bladder sections by fluorescence to determine where the labelled GAG was found.

## Methods

Texas Red-labelled chondroitin sulphate was prepared by mixing 10 μL Texas Red Hydrazide (Molecular Probes, Eugene, OR), 10 mM in dimethyl formamide, 40 μL of 1 mM chondroitin sulphate (Stellar Healthcare, London, Ontario), and 5 μL of 50% acetic acid, The chondroitin sulphate was assumed to be 20,000 molecular weight, yielding a concentration of 20 mg/ml as being equivalent to 1 mM. The reaction was allowed to occur overnight at room temperature. The next morning, the bond was reduced and made permanent by adding 10 μL of 150 mM sodium cyanoborohydride for 2 h. The labelled ChS was separated from unincorporated label by gel filtration on Biogel P-4.

Mice (C57BL/6NHsd, 9–10 weeks old) were anesthetized by intraperitoneal injection of ketamine 40 mg/kg and xylazine 5–10 mg/kg and catheterized with a 24 Ga × 3/4" intravenous catheter (Terumo Medical, Elkton, MD). Bladder damage was produced by 3 methods. (a) Protamine, 1 mg/ml, was instilled into the bladder until fullness and allowed to remain for 10 minutes. The protamine was then washed out with 3 fillings of buffered saline. (b) Dilute HCl, 10 mM was prepared by dilution in saline and was administered in the same way as described for the protamine except that a 0.1 M sodium bicarbonate wash was used as the first rinse;. (c) Trypsin, 1 mg/ml, was dissolved in saline and instilled into the mouse bladder where it was allowed to remain for 30 min. The trypsin was washed out with three fillings of buffered saline. Control animals were treated with buffered saline only. Three animals were in each group. The mouse bladders were then filled with Texas Red-labeled chondroitin sulphate, which was allowed to remain for 1 hr. The bladder was gently rinsed as described above. The animals were then euthanized by cervical dislocation with death being confirmed by opening the chest. The bladders were removed, flash frozen in OCT, and sections were obtained for fluorescence.

The bladders were sectioned (2 μm thick) with a cryostat and then examined first by ordinary fluorescence (2 sections per animal) and then by confocal microscopy using a Leica TCS NT confocal system (1 section). A representative field was selected for confocal microscopy. Texas Red emission was stimulated with excitation at 568 nm and the fluorescence was collected using a 630 nm bandpass filter. The nuclei were visualized by labelling with 20 μM Hoechst 33259. Sections were also stained with hematoxylin and eosin (H&E) and a low-pH Alcian Blue counterstained with Nuclear Fast Red. For analysis of the fluorescence intensities to assess relative binding, areas of urothelium in the image were outlined with the "lasso" tool of PhotoShop. Total pixels within the area were counted. The red colour was then detected within that area and the average brightness and number of pixels was calculated. The integrated intensity was calculated as the average pixel intensity × number of red pixels/total area in pixels. For the controls, the unenhanced image was used so that it would be comparable to the other images. Images were interpreted without knowledge of the treatment.

## Results

**Figure 1:**
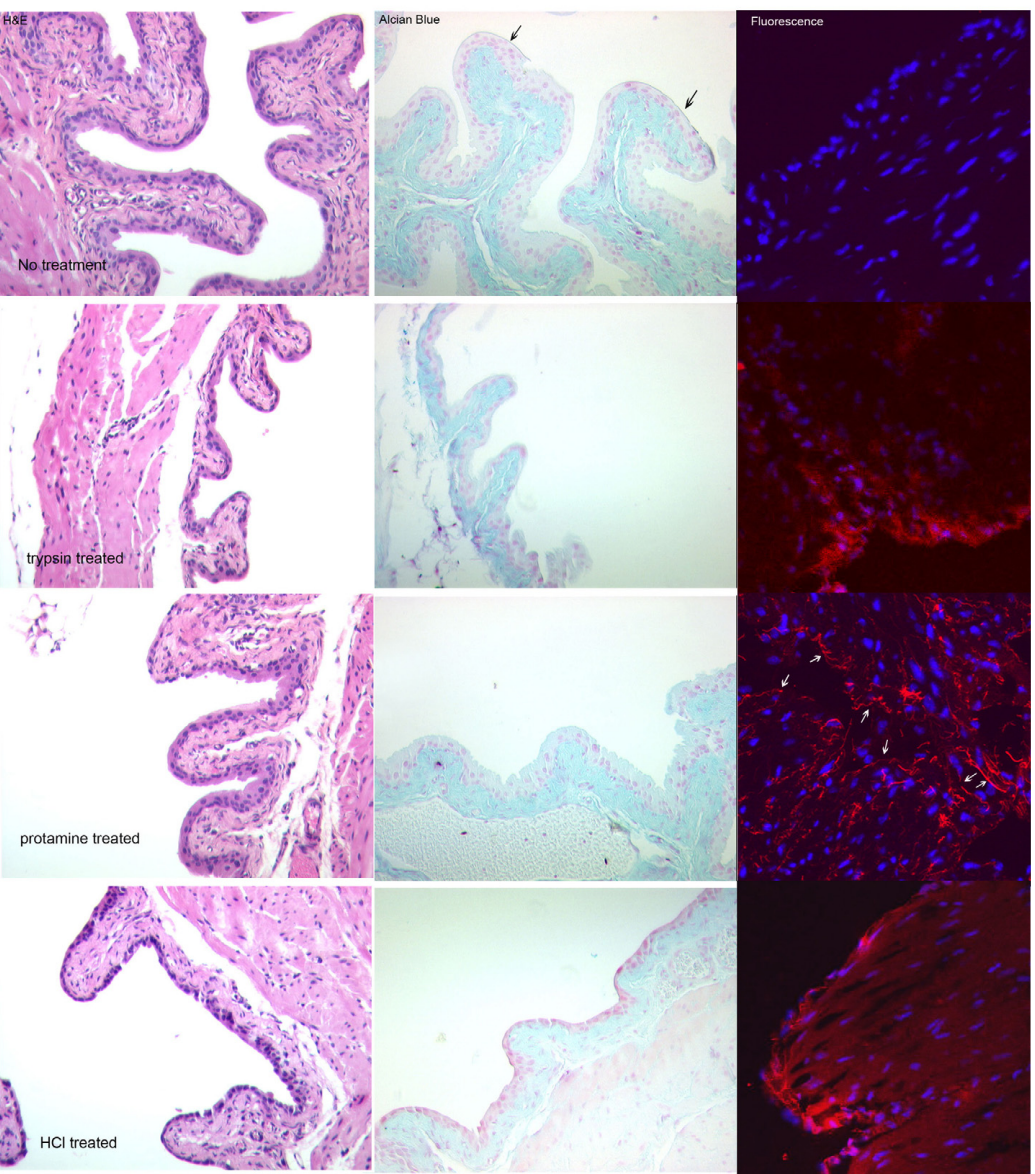
**Damage produced by each model of bladder damage and binding of Texas Red-labelled chondroitin sulphate to mouse bladder**. Images in the first two columns are transmitted light images of bladder sections stained with H&E or Acid Alcian Blue to demonstrate bladder damage. Acid Alcian Blue binds to glycosaminoglycans and therefore stains the connective tissue and the "GAG Layer," which is indicated by arrows in the control image. The fluorescence images are digitally combined images of Hoechst 33258 fluorescence (blue) to show nuclei and Texas Red-labelled chondroitin sulphate (red). Arrows have been added to the fluorescence image of the protamine-treated bladder to show the location of the urothelium. From top to bottom, the rows show the controls, bladders treated with 1 mg/ml trypsin (30 min), 1 mg/ml protamine (10 min) and 10 mM HCl (10 min.) respectively. The fluorescence image of the control was digitally enhanced to demonstrate the small amount of binding that occurred, but which was undetectable at the settings used for the other images (Table 1).

Figure 1 shows that each of the models of bladder damage produces a different pattern of damage. This figure shows both a conventional H&E stain (A) and an Alcian Blue stain (B) with the fluorescent images shown in (C). The controls showed an even urothelium with a well-formed endogenous "GAG layer" that appeared as a thin, blue line on the luminal surface of the urothelium. The luminal surface was even and showed the characteristic umbrella cells. The separation of the entire urothelium from the stroma seen in all the damaged bladders probably represents actual damage induced by, for example, edema resulting from loss of permeability rather than an artefact of sectioning. Trypsin removed most of the entire umbrella cell layer along with the "GAG layer." Protamine treatment produced little overt urothelial damage, but did roughen the luminal surface. The dilute HCl treatment also stripped off the umbrella cells and weakened the connection between urothelium and the stroma.

As shown in the fluorescence images, in control animals which experienced only saline instillation into the bladder, the label was found only on a narrow band corresponding to the luminal cell layer. The amount of TR-labeled chondroitin sulphate that actually bound to the controls was minimal, and the image shown in the figure was digitally enhanced to visualize the small amount that did bind. The dilute HCL damage model and the trypsin-damage model showed increased binding of the label to the bladder surface, but the binding still was confined mainly to the damaged urothelium. In the protamine damage model, the amount of binding was reduced. In no case, however, was significant penetration of the bladder by the label seen. Table 1 shows the quantitative image densitometry measurement of the amount of red fluorescence found at the urothelial surface of each of the images. Binding to the normal bladder surface was undetectable, but could be revealed by digital enhancement of the image. The trypsin- and HCl-damaged bladders bound the most labelled chondroitin sulphate, whereas the protamine damaged bladder bound about 1/5^th ^the amount bound by the other two models. These values are consistent with the level of damage observed.

**Table 1:** Comparison of binding of TR-labeled Chondroitin Sulphate to bladder urothelium in experimental bladder damage.

	Control	Trypsin	Protamine	Dil. HCl
Mean pixel intensity	0	139 ± 17	167 ± 24	156 ± 28
Red Area (pixels)	0	5,952	2,058	4,814
Urothelial Area (pixels)	366,025	81,879	79,623	64,528
Normalized Red	0	0.0727	0.0133	0.0746
Integrated Red	0	10.1 ± 1.3	2.2 ± 0.3	11.6 ± 2.1

## Discussion

Although large placebo responses have clouded clinical trials of replacement GAG therapy, thousands of patients have benefited from therapy with one of the agents used for allegedly replacing the bladder "GAG Layer." The mechanism of action could arise from physically coating the bladder surface and restoring impermeability as Parsons has claimed [19], or could be due to a more complex mechanism [20]. The normal urothelium has minimal affinity for chondroitin sulphate and binds it only on the luminal surface of the umbrella cells in miniscule quantities. However, the chondroitin sulphate did not penetrate into the deeper muscle layers of the bladder when the urothelium was damaged or destroyed, but bound extensively to the damaged bladder surface. Two of the bladder models yielded virtually identical results, but the pattern seen with protamine damage was different, and less chondroitin sulphate was bound than was observed in the trypsin and dilute HCl damage models. Lewis and co-workers [21] observed that protamine was inserted into the apical cell membrane, destroying the permeability barrier, but that glycosaminoglycans extracted the protamine, thereby restoring the bladder impermeability. Our findings are not inconsistent with this model, in which case both the extracted protamine and its complexed chondroitin sulphate would be removed with the fluid. The net result could be decreased binding of the label. However, the protamine instillation produced less overt damage as well.

The observed pattern of binding to damaged bladder suggests that its main action is on the urothelial surface and is not due to a pharmacologic effect. Both heparin and pentosanpolysulphate have pharmacologic effects on the coagulation system [22], whereas chondroitin sulphate, a normal constituent of the bladder surface, and hyaluronate do not [23]. All of these glycosaminoglycan molecules have effects on mast cells and other cells within the bladder at high concentrations *in vitro *[24-26]. The fact that all of these glycosaminoglycan molecules alleviate symptoms in some patients in spite of not penetrating into the bladder and remaining on the surface, even in the face of extensive damage, suggests a physical effect is responsible for clinical efficacy at least of chondroitin sulphate.

We suggest that one possible mechanism for the symptom-relieving effect of this class of compounds is they may exclude urinary proteases rather than salts. Theoretical calculations based on actual measurements of the amount of glycosaminoglycan found normally on the human bladder surface showed the presence of a dense layer of glycosaminoglycan will yield a bound water layer that reduces the concentration of salts at the bladder surface [20]. Lewis and Clausen earlier had demonstrated that urinary proteases degrade bladder ion channels [27], and Negrete and co-workers demonstrated the impermeability of rabbit bladder lay in the apical membrane [15]. Because the highly charged GAG layer with its attendant bound water layer would be a far more significant barrier for macromolecules than small, inorganic ions, we speculate that the GAG layer may normally function to exclude macromolecules rather than salts. Therefore the therapeutic benefit of exogenous glycosaminoglycans may result from exclusion of proteases or other macromolecules from the bladder surface where they could produce additional damage.

## Conclusion

Chondroitin sulphate instilled intravesically into three animal models of bladder damage coated the damaged bladder surface and failed to penetrate the bladder. This suggests they act at the bladder surface rather than in deeper layers of the bladder.

## Competing interests

This work was supported by a grant from Stellar Healthcare, Inc., a company that markets Uracyst for the treatment of Interstitial Cystitis.

## Authors' contributions

Jean Coffman obtained frozen sections and provided histologic interpretations. Kimberly Kyker performed the confocal microscopy and provided interpretations. Robert Hurst was responsible for the overall design of the experiments and their interpretation and for writing the manuscript.

## Pre-publication history

The pre-publication history for this paper can be accessed here:


